# Coronary artery to left ventricle fistula

**DOI:** 10.1186/1476-7120-3-35

**Published:** 2005-11-08

**Authors:** Angel López-Candales, Vivek Kumar

**Affiliations:** 1 Cardiovascular Institute at the University of Pittsburgh Medical Center, Pittsburgh, PA, USA

**Keywords:** Aortic valve disease, coronary fistulae, echocardiography, septal hypertrophy, myectomy

## Abstract

**Background:**

Coronary cameral fistulas are an uncommon entity, the etiology of which may be congenital or traumatic. They involve abnormal termination of a coronary artery, usually the right coronary, into a cardiac chamber, usually the right ventricle.

**Case Presentation:**

We describe a case of female patient with severe aortic stenosis and interventricular septal hypertrophy that underwent bioprosthetic aortic valve replacement with concomitant septal myectomy. On subsequent follow-up an abnormal flow traversing the septum into the left ventricle was identified and Doppler interrogation demonstrated a continuous flow, with a predominantly diastolic component, consistent with coronary arterial flow.

**Conclusion:**

The literature on coronary cameral fistulas is reviewed and the etiology of the diagnostic findings discussed. In our patient, a coronary artery to left ventricle fistula was the most likely explanation secondary to trauma to the septal perforator artery during myectomy. Since the patient was asymptomatic at the time of diagnosis no intervention was recommended and has done well on follow-up.

## Background

Communications between coronary arteries and cardiac chambers are likely congenital in origin. [1] However, in certain instances they might be acquired and is usually secondary to either trauma or after invasive cardiac procedures [2-8]. Physiologic derangements depend on the site of origin, size of the fistulae and on the receiving chamber [1,9-11]. It has been reported that the right coronary artery is the most likely site and the right ventricle the major receiving chamber [1,9]. We describe a case of female patient with severe aortic stenosis and interventricular septal hypertrophy that underwent bioprosthetic aortic valve replacement with concomitant septal myectomy. On subsequent follow-up and while asymptomatic, an abnormal continuous color flow signal with a predominant diastolic component, consistent with coronary arterial flow, traversing the septum into the left ventricle was identified.

## Case Report

A 74-year-old female with a history of severe aortic stenosis and interventricular septal hypertrophy underwent bioprosthetic aortic valve replacement with concomitant septal myectomy. Two months after the surgical intervention she presented to another hospital with syncope. On presentation, it was described that this obese patient was bradycardic with a heart rate of 40 beats per minute with stable blood pressure readings. No jugular venous distention was noted and occasional cannon A waves were noted. Examination of the lungs revealed adequate aeration in all fields with no crackles or wheezing. Point of maximum impulse was not displaced. Regular heart sounds with variable intensity were noted with no atrial or ventricular gallops but an early systolic murmur grade II/VI was described noted at the left sternal border. Due to the symptomatic bradycardia a dual chamber pacemaker was recommended and placed without complications. The patient was subsequently discharged home 24 hours after the pacemaker implantation. Four months post pacemaker implantation she was seen in follow-up and she doing fine and reported no complaints. An echocardiogram was obtained and it was reported that normal left ventricular systolic function as well as prosthetic aortic valve function were noted, with no other abnormalities.

**Figure 1:**
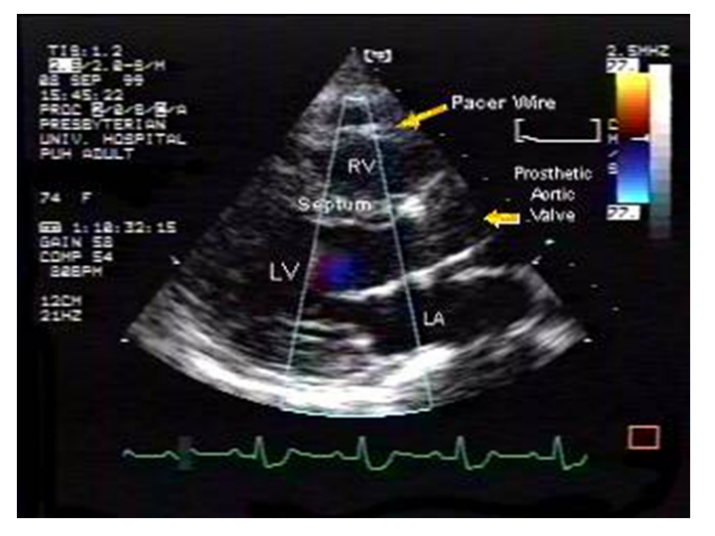
Parasternal long axis view showing an abnormal color flow signal arising from a thick interventricular septum with a predominant flow away from the transducer into the left ventricular cavity. A predominant diastolic component is shown. The position of the pacer wire and prosthetic aortic valve are also shown. (RV = right ventricle, LV = left ventricle, LA = left atria).

**Figure 2:**
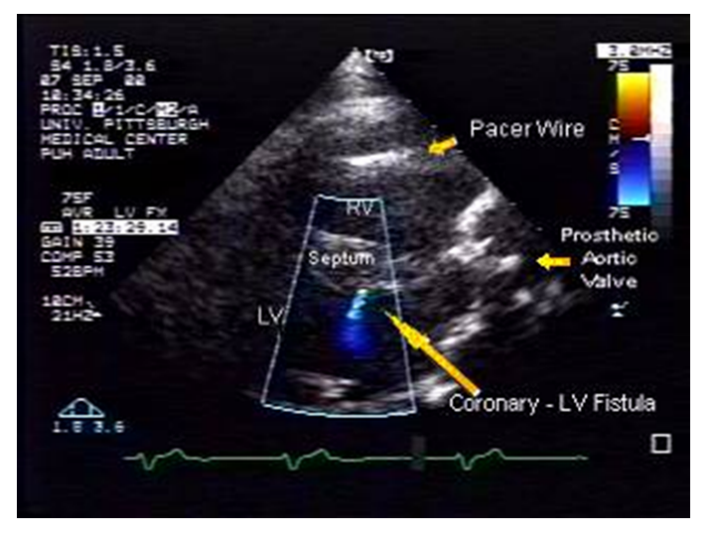
Similar parasternal image showing the predominantly diastolic color flow image component. (RV = right ventricle, LV = left ventricle, LA = left atria).

**Figure 3:**
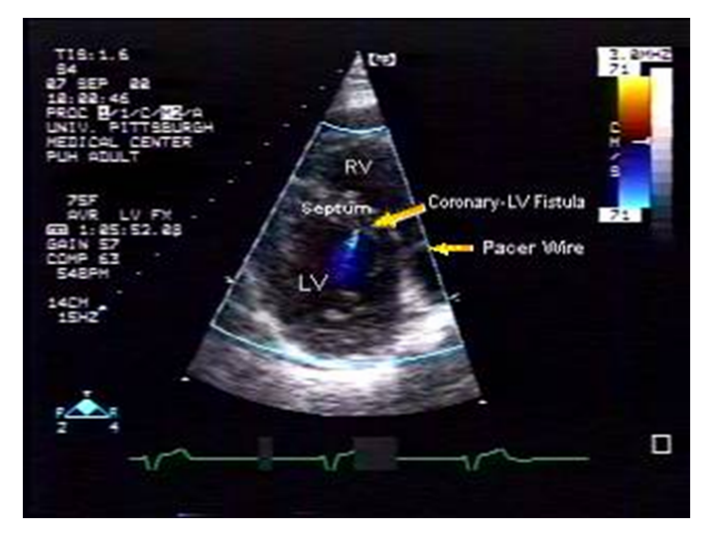
Short axis view showing the color flow signal with regards to the septum. (RV = right ventricle, LV = left ventricle, LA = left atria).

The patient then relocated and was seen a year later for the first time at our institution. An echocardiogram obtained at the time of her initial visit, while still asymptomatic, showed normal left ventricular chamber dimensions, systolic function, and bioprosthetic valve function. In addition, a pacer wire that was correctly positioned in the right ventricular apex was also seen. However, an abnormal color flow signal arising from the interventricular septum with a predominant flow away from the transducer into the left ventricular cavity was noted. Continuous and pulse wave Doppler interrogation demonstrated a continuous flow with a predominant diastolic component, as shown in Figures 1, 2 and 3, all these findings consistent with coronary arterial flow. This abnormal color flow signal, traversing the interventricular septum, was never identified in previous studies.

The patient presented in this case was asymptomatic at the time of diagnosis and consequently no intervention was recommended. The patient was seen in follow-up and was doing fine, reporting no complaints.

## Discussion

Communications between coronary arteries and cardiac chambers are often congenital malformations [1]. However, in certain instances they are acquired usually as a result from either trauma or after invasive cardiac procedures [2-8]. Physiologic derangements depend on the site of origin, size of the fistulae and on the receiving chamber [1,9-11]. The right coronary artery is the most likely site of origin in 55% of the cases while the left coronary artery system is involved in 35%. The major receiving chamber is the right ventricle (45%), right atrium (25%), pulmonary artery (15–20%) and less commonly in the coronary sinus (7%) [1,9]. In all reports, coronary cameral fistula least often drains into the left atrium or left ventricle. The size of the fistulae and the difference between the systemic and receiving chamber resistances determine the volume of the shunt. Regardless of these variables, flow moves from the coronary arteries to the lower pressure chambers. Most coronary artery fistulae are small and consequently myocardial blood flow is not compromised and the patient is usually asymptomatic. In some cases, however, coronary artery steal does occur with consequent development of ischemia in myocardial segments perfused by the coronary artery distal to the fistula [1,9-11].

Spontaneous closure has been reported in children but is less frequently noted in adults [12,13]. Spontaneous closure may be a more common occurrence in biopsy-related coronary cameral fistula.

A loud continuous murmur usually located at the lower sternal border identifies many patients with coronary artery fistula. In the case presented, a grade II/VI systolic murmur was documented in this obese patient.

Significantly enlarged coronary arteries can be detected by two-dimensional echocardiography. The actual diagnosis of a coronary artery fistula can often be made with transthoracic two-dimensional and color Doppler echocardiography in children. However, in adults, transesophageal echocardiography may be more sensitive [14-17]. Nowadays, the anatomic course and localization of coronary artery fistula can be made with either contrast-enhanced computer tomography with three-dimensional reconstruction or magnetic resonance imaging [18-21].

Hemodynamically significant fistula with a left to right shunt may lead to congestive heart failure, pulmonary artery hypertension, and myocardial ischemia by steal phenomenon with or without cardiac arrhythmias. The hemodynamic consequence of the coronary cameral fistula depends on the size of the fistula and the communicating chamber. Uncommon sequelae associated with this clinical entity include endocarditis, embolization of thrombotic material from the aneurysmal fistula, and potential rupture of the aneurysm [22]. Hemodynamically insignificant fistulae are clinically silent and if not associated with other abnormal findings usually require no further treatment. The risk of endocarditis and the need for endocarditis prophylaxis in untreated patients remains controversial. In contrast, large and hemodynamically significant fistulae should be closed by ligation [22-24]. Smaller coronary fistulae tend to get larger with age. As a result, it is usually recommended that elective closure be performed early in patients who have symptoms or who are asymptomatic but have a continuous murmur or a systolic murmur with an early diastolic component [22-24].

Given the characteristics of this case that involved the insertion of a bioprosthetic valve, myectomy and pacemaker insertions; each variable needed individual consideration. First, traumatic formation of a ventricular septal defect secondary to either surgical myectomy or during aortic valve replacement was a strong consideration. A defect was never identified; the residual interventricular septum was still thick, and most importantly the characteristic spectral signal of a ventricular septal defect had a predominant systolic flow component. Secondly, a trauma to the ventricular septum after pacemaker implantation could also account for this. Given the adequate position of the pacemaker lead in the right ventricular apex and our own previous description of a case reporting pacemaker trauma [25], this possibility appears unlikely. Alternatively, a septal defect could have been created at the time of the pacemaker insertion. However, absence of the characteristic echocardiographic features of a ventricular septal defect, as previously explained, made this possibility also improbable. Therefore, we postulate that this abnormal continuous flow, with a predominant diastolic component, was most consistent with coronary arterial flow. Consequently, given the location of this abnormal flow the most likely explanation was trauma to the septal perforator artery during myectomy resulting in a fistula into the left ventricle. The delayed clinical presentation might have been the result of an initial aneurysmal dilatation of the involved coronary artery with subsequent rupture and formation of the fistula.

## Conclusion

We describe a case of female patient with severe aortic stenosis and interventricular septal hypertrophy that underwent bioprosthetic aortic valve replacement with concomitant septal myectomy. On subsequent follow-up an abnormal flow traversing the septum into the left ventricle was identified and Doppler interrogation demonstrated a continuous flow, with a predominantly diastolic component, consistent with a coronary arterial flow. The literature on coronary cameral fistulas was reviewed and the etiology of the diagnostic findings discussed. In our patient, a coronary artery to left ventricle fistula was the most likely explanation secondary to a trauma to the septal perforator artery during myectomy. Since the patient was asymptomatic at the time of diagnosis no intervention was recommended and has done well on follow-up.

## List of abbreviations

None

## Competing interests

The author(s) declare that they have no competing interests.

## Authors' contributions

Dr. López-Candales interpreted the echocardiogram and Dr. Vivek and Dr. López-Candales prepared the manuscript and the literature reviewed. Both authors have approved the final review of the manuscript.
